# DNA Damage in Blood Leukocytes of Prostate Cancer Patients Undergoing PET/CT Examinations with [^68^Ga]Ga-PSMA I&T

**DOI:** 10.3390/cancers12020388

**Published:** 2020-02-07

**Authors:** Sarah Schumann, Harry Scherthan, Torsten Frank, Constantin Lapa, Jessica Müller, Simone Seifert, Michael Lassmann, Uta Eberlein

**Affiliations:** 1 Department of Nuclear Medicine, University of Würzburg, Oberdürrbacher Str. 6, 97080 Würzburg, Germany; 2 Bundeswehr Institute of Radiobiology affiliated to the University of Ulm, Neuherbergstraße 11, 80937 München, Germany; 3 Department of Nuclear Medicine, University of Augsburg, Stenglinstraße 2, 86156 Augsburg, Germany

**Keywords:** DNA double-strand breaks, γ-H2AX, 53BP1, nuclear medicine, dosimetry, Ga-68, PSMA, PET/CT, contrast agent, prostate cancer

## Abstract

The aim was to investigate the induction and repair of radiation-induced DNA double-strand breaks (DSBs) as a function of the absorbed dose to the blood of patients undergoing PET/CT examinations with [^68^Ga]Ga-PSMA. Blood samples were collected from 15 patients before and at four time points after [^68^Ga]Ga-PSMA administration, both before and after the PET/CT scan. Absorbed doses to the blood were calculated. In addition, blood samples with/without contrast agent from five volunteers were irradiated ex vivo by CT while measuring the absorbed dose. Leukocytes were isolated, fixed, and stained for co-localizing γ-H2AX+53BP1 DSB foci that were enumerated manually. In vivo, a significant increase in γ-H2AX+53BP1 foci compared to baseline was observed at all time points after administration, although the absorbed dose to the blood by ^68^Ga was below 4 mGy. Ex vivo, the increase in radiation-induced foci depended on the absorbed dose and the presence of contrast agent, which could have caused a dose enhancement. The CT-dose contribution for the patients was estimated at about 12 mGy using the ex vivo calibration. The additional number of DSB foci induced by CT, however, was comparable to the one induced by ^68^Ga. The significantly increased foci numbers after [^68^Ga]Ga-PSMA administration may suggest a possible low-dose hypersensitivity.

## 1. Introduction

PET/CT with ^68^Ga-labelled prostate-specific membrane antigen (PSMA) is a non-invasive diagnostic technique to image prostate cancer patients with increased PSMA expression [1]. In recent years, several ligands such as [^68^Ga]Ga-PSMA-11, [^68^Ga]Ga-PSMA-617, and [^68^Ga]Ga-PSMA I&T have been developed for PET/CT diagnostics, demonstrating similar biodistribution and imaging properties [1]. Among others, [^68^Ga]Ga-PSMA I&T is of interest, as it can also be labelled with ^177^Lu for therapeutic purposes [2,3].

[^68^Ga]Ga-PSMA PET/CT examinations deliver rather low effective doses [1,2]. Nevertheless, the ionizing radiation applied may potentially cause DNA double strand breaks (DSBs) whose misrepair may increase the risk of secondary malignancies. Recently we analyzed, for the therapeutic PSMA ligand [^177^Lu]Lu-PSMA I&T, the time- and absorbed dose-dependent induction and repair of DSBs with a DNA damage focus assay that utilizes immunofluorescent staining with antibodies for γ-H2AX and 53BP1 DNA DSB damage markers [4,5,6] and the analysis of co-localized γ-H2AX+53BP1 foci in the cell nuclei of peripheral blood leukocytes of prostate cancer patients to assess DSB damage [7]. The time-dependency of the DSB induction is characterized by a linear increase of the number of DSB foci in the first few hours after administration of the radiopharmaceutical, followed by a phase of equilibrium between repair and induction of DSBs, and subsequently by predominant repair leading, in most patients, to a complete decline to baseline foci levels [7,8,9]. Our group applied this DSB damage assay successfully in a number of ex vivo and in vivo studies with different therapeutic radionuclides such as ^131^I, ^177^Lu and the radium isotopes ^223^Ra and ^224^Ra [7,8,9,10,11,12,13].

Up to now, there are few studies on the formation of DNA DSB damage after the diagnostic application of radiopharmaceuticals. However, the relationship between damage induction and the absorbed dose has not been investigated so far, making the results of these studies difficult to compare. Besides three studies concentrating on cardiac imaging [14,15,16], the focus of these biodosimetry studies is mainly on effects after [^18^F]FDG PET(/CT) examinations: Using individual γ-H2AX foci enumeration, May et al. investigated the formation of DSBs in blood lymphocytes of patients undergoing [^18^F]FDG PET/CT examinations [17]. The authors reported a correlation between the CT-induced DSB foci and the dose-length-product, while patient-specific dosimetry was absent. Schnarr et al. measured γ-H2AX fluorescence intensity before and after [^18^F]FDG PET scans and observed a variable response between the patients [18]. Using γ-H2AX fluorescence intensity measurements, Prasad et al. failed to reveal a significant increase of the number of induced DSBs in patients undergoing [^18^F]FDG PET/CT examinations [19]. Nautiyal et al. used the comet assay and the micronucleus assay to investigate DNA damage in patients after [^18^F]FDG PET/CT scans, focusing only on the difference between non-contrast and contrast-enhanced examinations while data on the dose-dependency of the induced damage were absent [20].

As this is the first study with patients undergoing [^68^Ga]Ga-PSMA PET/CT examinations and no studies on the correlation between DSBs and the absorbed dose to the blood for diagnostic radiopharmaceuticals have been published so far, the present study serves a three-fold purpose: (i) To study the induction of DNA damage after irradiation with the high energy positron emitter ^68^Ga, (ii) to establish a link between the number of DSBs and the absorbed doses to the blood in the very low-dose range of <10 mGy, and (iii) to quantify the influence of the CT and the iodinated contrast agent on the induction of DSBs.

## 2. Results

### 2.1. In Vivo Study

#### 2.1.1. Patients and Blood Sampling

Fifteen patients (P1–P15) aged between 56 and 82 years (average: 71 ± 6 years) were enrolled in this study. The mean administered activity was (140 ± 21) MBq of [^68^Ga]Ga-PSMA. All patients except one (P7) showed PSMA uptake. The displayed computed tomography dose index (CTDI_vol_) of the whole-body CT was (14.3 ± 2.9) mGy in average. For the low-dose CT of the thorax the CTDI_vol_ was 6.1 mGy for all patients. The demographic, clinical, and scan-related data of all patients are listed as Supplementary Material in Table S1.

Blood samples for the time points t_1_, t_2_, t_3_, and t_4_ were taken (18 ± 5) min, (56 ± 10) min, (90 ± 16) min and (185 ± 15) min after administration. The sample at t_2_ for patient P8 could not be taken and is therefore missing. The sample at t_2_ of patient P7 and the sample at t_3_ of patient P9 could not be evaluated. In average, there were (8 ± 3) min between the blood withdrawal and the start of the processing of the samples, i.e., the isolation of the leukocytes. The time difference between the CT scan and the start of the processing of the blood samples averaged (19 ± 5) min. For dosimetry calculations, always the start time of the processing of the samples was used, which was (24 ± 5) min, (64 ± 11) min, (101 ± 14) min, and (193 ± 15) min after administration for t_1_, t_2_, t_3_, and t_4_, respectively.

#### 2.1.2. Dosimetry

For describing the activity retention in the blood, biexponential fit functions were used assuming only physical decay for the second component. For describing the activity retention in the whole body, monoexponential fit functions were used. 

The average absorbed doses to the blood resulting from ^68^Ga application at the time points t_1_, t_2_, t_3_, and t_4_ were (1.0 ± 0.3) mGy, (1.8 ± 0.5) mGy, (2.1 ± 0.6) mGy, and (2.6 ± 0.7) mGy, respectively. At t_2_, the absorbed doses to the blood resulting from ^68^Ga application was below 3 mGy in all patients; at t_4_, it was less than 4 mGy. Total absorbed doses to the blood (*t* = ∞) were not calculated because of the limited number of sampling time points and the fact that the additional contribution of the CT for all time points ≥ t_3_ could not be considered.

**Figure 1:**
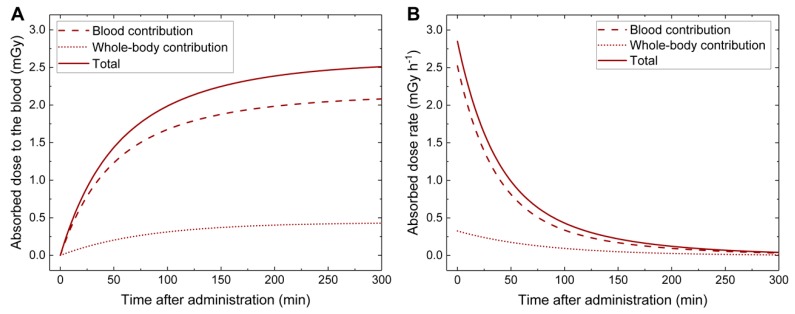
In vivo study—time-dependency of the absorbed dose to the blood and the absorbed dose rate. (**A**) Absorbed dose to the blood as a function of time for a selected patient (P14) with average fit parameters. For this graph, only the absorbed dose to the blood due to ^68^Ga administration is considered and the contribution of the computed tomography (CT) has not been taken into account. The contribution of the blood is shown as a dashed line whereas the whole-body contribution is shown as a dotted line. The solid line depicts the total absorbed dose to the blood that is the sum of both contributions. (**B**) The corresponding absorbed dose rates as function of time for the same patient.

The time-dependency of the absorbed dose to the blood resulting from ^68^Ga application according to Equation (4) is shown for a selected patient (P14) with average fit parameters in Figure 1A. The corresponding time-dependency of the absorbed dose rate, defined as the derivative of Equation (4) over time, is shown in Figure 1B for the same patient.

#### 2.1.3. Time- and Absorbed Dose-Dependency of γ-H2AX+53BP1 Foci

**Figure 2:**
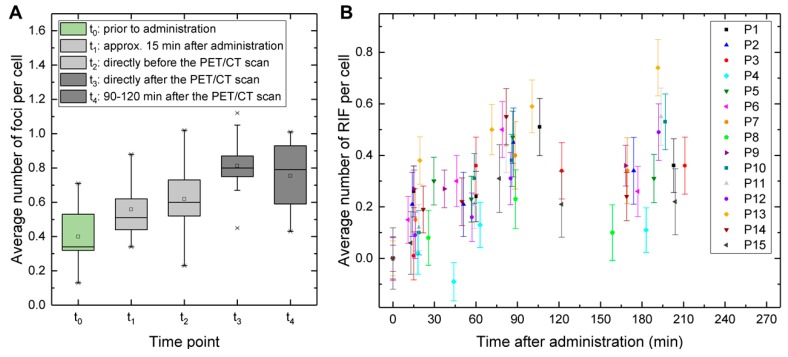
In vivo study—time-dependency of the average number of foci and radiation induced foci (RIF). (**A**) Boxplot of the average number of foci per cell at the five sampling time points t_0_ to t_4_. The mean of the average number of foci at the time points t_1_, t_2_, t_3_, and t_4_ is significantly elevated compared to the mean baseline value at t_0_. A significant increase of the average number of foci was also observed from t_1_ to t_2_ and from t_2_ and t_3_. (**B**) Patient-specific average number of RIF per cell as a function of the time after administration.

The mean of the average number of baseline DSB foci per cell in the t_0_-samples was 0.40 ± 0.17. The mean of the average number of foci per cell at the time points t_1_, t_2_, t_3_, and t_4_ was 0.56 ± 0.16, 0.62 ± 0.20, 0.81 ± 0.16, and 0.75 ± 0.19, respectively (Figure 2A). For all four time points, it was significantly elevated compared to the baseline value at t_0_. A significant increase of the mean of the average number of foci was also observed from t_1_ to t_2_ and from t_2_ to t_3_. From time point t_3_ to time point t_4_, the average number of DSB foci per cell remained constant within the range of the counting error in eleven of the patients. A clear increase was only observed in one patient (P12) while a clear decrease was observed in two patients (P6 and P14). For P9, an evaluation was not possible due to the missing t_3_-sample. The patient-specific time-dependency of the average number of radiation induced foci (RIF) per cell is shown in Figure 2B.

**Figure 3:**
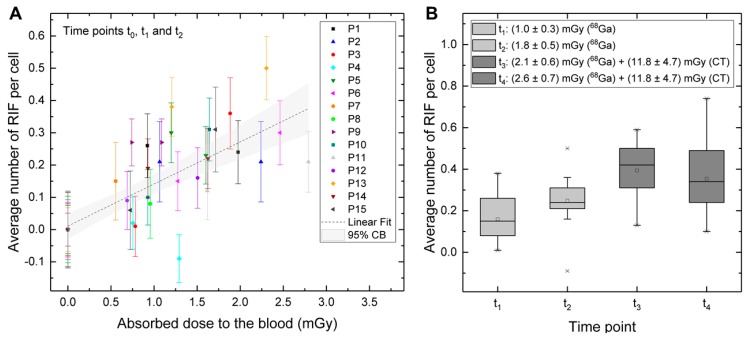
In vivo study—absorbed dose-dependency of the average number of RIF. (**A**) Average number of RIF per cell as a function of the absorbed dose to the blood (time points t_0_, t_1_ and t_2_) with a linear fit to the pooled data, including a 95% confidence band. (**B**) Boxplot of the average number of RIF per cell at the sampling time points t_1_ to t_4_ with the mean absorbed doses to the blood stated in the legend.

The average number of RIF per cell as a function of the absorbed dose to the blood for the time points before the CT (only time points t_0_, t_1_ and t_2_) is shown in Figure 3A. In the corresponding dose range for the t_1_-samples and the t_2_-samples, from 0.5 mGy to 2.8 mGy, an increasing number of RIF was observed. A linear fit to the pooled data resulted in the equation:

Average RIF per Cell = (0.130 ± 0.018) mGy^−1^ D_bl, Ga-68_ + (0.011 ± 0.020); R^2^ = 0.56 (1)

Figure 3B shows a boxplot of the average number of RIF per cell at the sampling time points t_1_ to t_4_ with the mean absorbed doses to the blood stated in the legend.

### 2.2. Ex Vivo Study

#### 2.2.1. Absorbed Dose-Dependency of γ-H2AX+53BP1 Foci

The measured absorbed dose values D_bl, CT_ were (10.6 ± 0.5) mGy, (20.3 ± 1.1) mGy, and (31.2 ± 2.1) mGy in average for the three different CT settings D1, D2, and D3, respectively.

**Figure 4:**
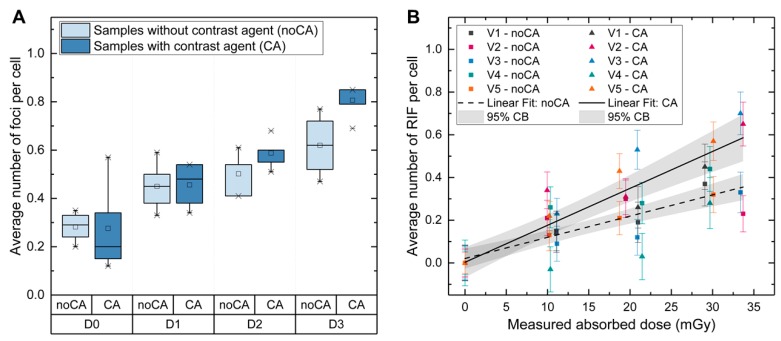
Ex vivo study—absorbed dose- and contrast agent-dependency of the average number of foci and RIF. (**A**) Boxplot of the average number of foci per cell after ex vivo irradiation with different CT settings resulting in the absorbed doses D0 (no irradiation), D1 (10.6 ± 0.5) mGy, D2 (20.3 ± 1.1) mGy and D3 (31.2 ± 2.1) mGy. (**B**) Average number of RIF per cell as a function of the measured absorbed dose for the five volunteers V1-V5 with linear fits including 95% confidence bands.

The mean of the average number of baseline foci per cell in the non-irradiated D0-sample was 0.28 ± 0.06 in the sample without contrast agent (noCA) and 0.28 ± 0.18 in the sample with contrast agent (CA). At D1, D2, and D3, the mean of the average number of foci per cell was 0.45 ± 0.10 (noCA) and 0.46 ± 0.09 (CA), 0.50 ± 0.09 (noCA) and 0.59 ± 0.06 (CA), and 0.62 ± 0.13 (noCA) and 0.81 ± 0.07 (CA), respectively (Figure 4A). However, it is to note that, unlike all other values, the CA-values at D3 were not normally distributed. The median value of the average number of foci per cell was 0.85 (min.: 0.69; max.: 0.85) in this case. To test for differences between the respective CA and the noCA groups, we applied paired t-tests. The differences between the two groups were not significant, except for the highest dose D3. As the values of D3-CA were not normally distributed, we also performed a Wilcoxon test, which was not significant.

The average number of RIF per cell as a function of the measured dose is shown in Figure 4B. Linear fits to the pooled volunteer data were performed for the noCA-samples and the CA-samples separately, resulting in the following linear equations:

Average RIF per Cell (noCA) = (0.010 ± 0.001) mGy^−1^ D_bl, CT_ + (0.021 ± 0.022); R^2^ = 0.78 (2)

Average RIF per Cell (CA) = (0.017 ± 0.002) mGy^−1^ D_bl, CT_ + (0.003 ± 0.034); R^2^ = 0.79 (3)

Applying Akaike information criteria (AIC) and an F-test verified that the two slopes are statistically different.

#### 2.2.2. Estimation of the CT-Caused Absorbed Dose to the Blood in the Patient Study

Based on the ex vivo calibration curve for the samples with CA (Equation (3), Figure 4B), the contribution of the CT to the absorbed dose to the blood in the patient study was estimated at D_bl, CT_ = (11.8 ± 4.7) mGy (Figure 3B). For this estimation, the simplifying assumption was made that the difference in the average number of RIF between time point t_2_ and time point t_3_ (ΔRIF_3→2_) was exclusively due to the radiation of the CT and that the contribution of the ^68^Ga can be neglected because of its very low absorbed dose rate at the time of the CT scan (as shown in Figure 1B). ΔRIF_3→2_ was inserted in Equation (3) for each patient separately and individual CT-doses were calculated. In two patients (P3 and P15) a decrease in RIF after the CT was observed (i.e., ΔRIF_3→2_ < 0), which would imply negative CT-doses. These patients were excluded from the analysis. Furthermore, three patients with missing t_2_- or t_3_-samples (P7, P8, and P9) had to be excluded from the analysis. In all other patients, ΔRIF_3→2_ was positive and the calculated CT-induced absorbed doses to the blood ranged from 3.9 mGy up to 19.2 mGy.

## 3. Discussion

In the current study, the γ-H2AX+53BP1 DSB focus assay was used to investigate DSB induction and repair in blood leukocytes of patients undergoing PET/CT examinations with [^68^Ga]Ga-PSMA I&T and to find a correlation to the absorbed dose to the blood. We observed an increased number of foci compared to the baseline values even at absorbed doses to the blood in the range between 0.5 mGy and 2.8 mGy.

The average number of RIF per cell in this very low-dose range was higher than expected according to a previously established ex vivo calibration curve for the internal irradiation of blood with the radionuclides ^131^I and ^177^Lu [10]. The slope of the latter ex vivo calibration curve was (0.0147 ± 0.0006) mGy^−1^ [10], similar to the slopes of in vivo calibration curves established in several patient studies investigating DSB induction during therapy with ^131^I (slope of (0.0117 ± 0.0006) mGy^−1^) [8], [^177^Lu]Lu-DOTATATE/DOTATOC (slope of (0.0127 ± 0.0009) mGy^−1^) [9] and [^177^Lu]Lu-PSMA I&T (slope of (0.0122 ± 0.0015) mGy^−1^) [7]. However, all our previous studies [7,8,9,10] mainly covered the dose range between 6 mGy and 100 mGy. Compared to these results, the slope in the present study of (0.130 ± 0.018) mGy^−1^ was approximately ten-fold higher, indicating that DSB induction might be increased in the very low-dose range <10 mGy, indicative of low-dose radio hypersensitivity. It is of note, however, that the evaluation of γ-H2AX+53BP1 foci in this very low-dose range is more error-prone than in higher dose ranges regarding its sensitivity, since the counting errors increase with a decreasing number of events.

An approximately ten times steeper slope (0.10 foci per mGy) of dose-dependent γ-H2AX foci in the low-dose region (below 10 mGy) was also reported by Beels et al., however, for blood samples irradiated with X-rays but not for samples that were exposed to ^60^Co γ-rays [21]. A similar low-dose hypersensitive response is also described in two other studies published by Beels et al. [22,23]. The authors depict a biphasic behavior of the dose response: A steep linear increase in γ-H2AX foci up to 6 mGy [22] or 10 mGy [23], followed by more flat linear increase at higher doses. In a study by Vandevoorde et al. a low-dose hypersensitivity has also been observed when using the quantification of γ-H2AX foci to investigate DNA damage induced by CT X-rays in pediatric patients [24]. Despite very low blood doses between 0.14 mGy and 8.85 mGy, there was a statistically significant increase of a median of 0.13 foci per cell after the examinations, which was considerably higher than expected from the extrapolation of high-dose behavior based on a linear-no-threshold hypothesis [24]. One possible explanation for the increased number of DSBs for very low doses are bystander effects in cells that were not directly hit [25]. Alternatively, the relatively high amount of DSBs could also be related to a reduced repair efficacy at very low doses, as reported in several studies investigating DNA DSB repair after low-dose X-ray irradiation of cultured human fibroblasts and mouse tissues [26,27,28]. This would lead to enrichment and persistence of foci induced in this dose range.

Up to now, there is no other patient study correlating DNA damage to absorbed doses to the blood in this diagnostic low-dose range, but there are few other studies [17,18,19] investigating DSB formation after [^18^F]FDG application reporting varying outcomes. The first study investigating formation and repair of PET/CT-induced DSBs in vivo was conducted by May et al. [17]. They analyzed γ-H2AX foci in 33 patients receiving 138-354 MBq [^18^F]FDG and one or two diagnostic CT scans and report that a median of 0.11 foci per cell and 0.17 foci per cell was induced by the [^18^F]FDG and the whole-body CT, respectively. The considerable higher amount of foci induced by the CT is in good agreement with our study, as we observed a median increase of 0.15 foci per cell (18 ± 5) min after [^68^Ga]Ga-PSMA I&T administration and a median increase of 0.21 foci per cell when comparing the foci number directly after the CT (t_3_) to the foci number directly before the CT (t_2_). However, since a different radiopharmaceutical was used and no dosimetry was performed by May et al. the results are difficult to compare. Furthermore, the different protocol for leukocyte isolation and fixation and the fact that, in our study, co-localizing γ-H2AX+53BP1 foci were evaluated, also impedes a direct comparison of foci numbers. In addition, May et al. observed a significant correlation between the CT-induced DSBs and the dose-length-product. In the present study, there was no significant correlation.

In contrast to May et al. other authors, who measured γ-H2AX fluorescence intensity in patients’ lymphocytes both before and after [^18^F]FDG application, reported that they did not find significant changes [18,19]. Schnarr et al. observed a varied response among ten patients when measuring γ-H2AX fluorescence intensity by flow cytometry before and after [^18^F]FDG PET scans [18]. Prasad et al. also used flow cytometry to measure γ-H2AX fluorescence intensity in the lymphocytes of 25 patients and reported an increase in the signal after the PET/CT scan that was, however, not significant because of a high inter-individual variation [19]. This indicates that the evaluation of γ-H2AX fluorescence intensities might not be sensitive enough to assess DNA damage after exposures to very low absorbed doses and that, as shown in our current study, the quantification of DSB foci seems to be a more suitable approach in this dose range.

In a recent study, Nautiyal et al. investigated the effect of ionizing radiation in patients undergoing non-contrast and contrast-enhanced [^18^F]FDG PET/CT examinations using both comet assay and micronucleus assay [20]. They did not investigate the dose-dependency of the induced DNA damage either, but focused on the difference between a patient group that received a contrast agent and a corresponding non-contrast group. As a result, they report a significant increase in DNA damage in the contrast group compared to the non-contrast group [20], which is in accordance with the observations made in the present ex vivo study.

Many further studies used the γ-H2AX assay both ex vivo and in vivo for investigation of DNA damage in blood lymphocytes induced during CT examinations and, in particular, to investigate the effect of iodinated contrast agents [23,29,30,31,32,33,34,35,36]. In agreement with the results of our ex vivo study, the majority of these studies report an increase of DNA damage in the contrast media-enhanced samples. Despite the findings in some of the ex vivo studies that this increase is only significant for high concentrations of the iodinated contrast agents used (up to 50 mg mL^−1^) or high radiation doses (up to 1 Gy) but not in a range that is clinically relevant for diagnostic procedures [30,34], many patient studies show that there is an 30 to 107% increase in DNA damage when contrast agents are applied compared to non-contrast enhanced examinations [31,32,34,35,36].

In our ex vivo study, we matched the CT settings and the concentration of the contrast agent as closely as possible with parameters comparable to the patient examinations. The absorbed dose range was chosen to be between 10 mGy and 30 mGy and the contrast agent concentration was chosen to be 5.55 mg mL^−1^ iodine concentration in the blood. A tendency of an increased amount of DNA DSBs was observed for all absorbed doses. Comparing the slope values of the ex vivo calibration curves established in this study, the slope for the CA-samples was 1.7 times higher than the slope for the noCA-samples. The increase of DNA damage after the application of iodinated contrast agents could be caused by increased energy deposition, as suggested by Harbron et al. who performed Monte Carlo modelling to obtain dose enhancement factors (DEFs) to blood for different blood vessel models and iodine concentrations [37]. For an iodine concentration of 5.0 mg mL^−1^, (which is similar to our experiments) they calculated a DEF of 1.63 for the intraluminal part of an artery model (120 kV tube voltage) [37]. Jost el al. also calculated DEFs for the same iodine concentration of 5.0 mg mL^−1^ and report a DEF of 1.56 [30]. The results of both groups are in excellent agreement with the experimental findings of our current study that suggest a DEF of 1.7.

Comparing the contribution of the CT to the absorbed dose to the blood in patients, which was (11.8 ± 4.7) mGy in average, with the contribution of the ^68^Ga leads to the conclusion that, for this hybrid imaging setup, the major contribution to the absorbed dose to the blood is caused by CT. However, the assessment of the CT-caused absorbed dose to the blood based on our ex vivo experiments is a simplification with some limitations: First, it is not possible to ensure that each t_3_-sample was processed at the exact time when the maximum of RIF induced by the CT was reached. The average time difference between the CT scan and the processing of the blood samples was (19 ± 5) min, which generally matches the observation that the maximum of γ-H2AX+53BP1 foci is reached 15 min to 30 min after external irradiation [4,6,38]. However, a variable time difference can be the reason for the varying number of RIF after the CT. Especially the decrease in RIF after the CT in patient P3 may be ascribed to insufficient time for maximum foci induction, since this was the patient with the minimum time difference between the CT scan and the blood sample processing of only 9 min. Aside from that, DSB repair kinetics are known to vary between individual patients [7,8,9]. Second, we cannot exclude that there is still DSB induction due to [^68^Ga]Ga-PSMA uptake, despite the low absorbed dose rate to the blood at the time of the CT scan (see Figure 1B). Third, in our ex vivo study we only covered the dose range between 10 mGy and 30 mGy, since this was the dose range anticipated from the CTDI_vol_ values of a whole-body CT with the settings used in this study. However, we cannot exclude completely that the number of RIF in the low-dose range may be higher than expected from the extrapolation, as observed after low-dose ^68^Ga exposure. This could have led to an overestimation of the CT-caused absorbed dose to the blood. The fact that the estimated value for the CT-caused absorbed dose to the blood is well in line with the average displayed CTDI_vol_ of the whole-body CT of (14.3 ± 2.9) mGy renders an overestimation, however, rather unlikely.

## 4. Materials and Methods

### 4.1. Ethics Committee Approval and Patient Consent

The research plan was presented to the Ethics Committee of the Medical Faculty of the University of Würzburg, Germany. The Ethics Committee approved the study stating that there were no objections to the conduct of the study (Az: 209/14 and 165/14). All procedures performed in studies involving human participants were in accordance with the ethical standards of the Ethics Committee of the Medical Faculty of the University of Würzburg and with the principles of the 1964 Declaration of Helsinki and its later amendments or comparable ethical standards. Informed consent was obtained from all individual participants included in the study.

### 4.2. In Vivo Study

#### 4.2.1. Patients

For the in vivo study, 15 prostate cancer patients (P1–P15) referred to our center for diagnostic PET/CT examinations were enrolled. The indications for the examinations were primary or re-staging, PSA-relapse or progressive disease. 

All patients received a standard activity of 150 MBq of [^68^Ga]Ga-PSMA I&T (SCINTOMICS GmbH, Munich, Germany) that was intravenously administered approximately one hour before the PET/CT scan (Biograph mCT, Siemens Healthineers, Erlangen, Germany). The radiolabeling process and quality control of the radiopharmaceutical was performed according to our standard operation procedure analogous to the method described by Herrmann et al. [2]. As a standard procedure, all patients received orally 10 mg furosemide (Lasix^®^, Sanofi-Aventis, Frankfurt, Germany) to reduce the absorbed dose to the bladder and 30 mL Peritrast^®^ (Dr. Franz Köhler Chemie GmbH, Bensheim, Germany) to enhance the CT contrast of the intestinal tract. Directly before the CT examination, 1 mL per 1 kg body weight of an iodinated contrast agent (Ultravist^®^-370, Bayer, Leverkusen, Germany) was administered intravenously. CT scans were performed at 120 kV with 210 mAs reference and an anatomy-based tube current modulation (Care Dose). Additionally, a low-dose CT of the thorax (120 kV, 80 mAs) was performed.

#### 4.2.2. Blood Sampling and External Dose Rate Measurements

Blood samples were taken prior (t_0_; for the determination of the number of individual baseline foci) and at four time points after the administration of [^68^Ga]Ga-PSMA I&T. The nominal sampling time points were: approximately 15 min after administration (t_1_), directly before the PET/CT scan (t_2_; approx. 60 min after administration), directly after the PET/CT scan (t_3_), and approx. 90–120 min after the PET/CT scan (t_4_). Li-Heparin blood collecting tubes (S-Monovette^®^, Sarstedt, Nümbrecht, Germany) were used for the blood withdrawals. 

In order to determine the contribution of the γ-radiation to the absorbed dose to the blood, 3–4 external dose rate measurements of each patient were performed after the administration of the activity, usually directly before or directly after each blood withdrawal. For this, a dose rate meter (6150AD^®^, automess GmbH, Ladenburg, Germany) was used to measure the dose rate in 1 m distance from the patient. The value of the first measurement that was performed prior to the first post-administration void or defecation by the patient, was decay corrected to the time of administration and normalized to 1, corresponding to 100% uptake.

#### 4.2.3. Activity Quantification

For activity quantification, an aliquot of each blood sample was measured in a calibrated high-purity germanium detector (Canberra, Rüsselsheim, Germany) and the γ-emission line at 511 keV (emission probability of 178%) was evaluated. The counting efficacy of the germanium detector was ascertained by measuring several NIST- and NPL-traceable standards. The measured number of counts at 511 keV was decay-corrected to the start time of the measurement. Then, the corresponding activity value was decayed to the time point of the blood withdrawal.

#### 4.2.4. Calculation of the Absorbed Doses to the Blood Related to ^68^Ga Application

Biexponential and monoexponential fit functions were used to model the time curves for the activity retention in the blood and in the whole-body, respectively, in each patient. Time-integrated activity coefficients (TIACs) for the activity concentration in the blood (τ_mL of bl_ (t) in hours per mL) and for the whole-body (τ_wb_ (t) in hours) were attained by integrating the time-activity fit functions over time, up to the time point t when the processing of the blood samples (i.e., the isolation of the leukocytes) started.

The contribution of the ^68^Ga to the absorbed dose to the blood D_bl, Ga-68_ (t) as a function of time was calculated according to the Medical Internal Radiation Dose (MIRD) formalism [39], in analogy to Eberlein et al. [9], using the following equation:

D_bl, Ga-68_ (t) = A_0_ (425 Gy mL GBq^−1^ h^−1^ · τ_mL of bl_ (t) + 0.0459 Gy kg^2/3^ GBq^−1^ h^−1^ · wt^−2/3^ τ_wb_ (t)) (4)

Here, A_0_ is the administered activity in GBq and wt is the weight of the patient in kg. To calculate the S-value for the self-irradiation of the blood, it was assumed that all β-energy is deposited in the blood (emission data taken from [40]), neglecting the contribution of the γ-radiation. For the calculation of the whole-body contribution, only the γ-radiation was considered and the weight-adapted S-value was taken from OLINDA/EXM [41]. A more detailed derivation of Equation (4) is given in the Supplementary Material of reference [9].

### 4.3. Ex Vivo Study

#### 4.3.1. Blood Sampling and Irradiation

For the ex vivo study, five healthy volunteers (V1–V5) aged between 23 and 61 years were included in the study. A total of 36 mL blood was taken from each volunteer using Li-Heparin blood collecting tubes (S-Monovette^®^, Sarstedt). The blood was subsequently split into eight 4.5 mL aliquots and pipetted into 5 mL round bottom tubes (Sarstedt). Directly before the irradiation, four of the aliquots were supplemented with 67.5 µL contrast agent (Ultravist^®^-370, Bayer), while the same amount of phosphate buffered saline (PBS) was added to the other four samples. The concentration of the contrast agent (15 µL per mL blood) was chosen to match the concentration in patients’ blood assuming a blood volume of 5 L for a patient of 75 kg bodyweight.

For the irradiation in the CT scanner, one blood sample with contrast agent (CA) and one blood sample without contrast agent (noCA) were placed at defined positions on the patient bench. All CT parameters were chosen according to the patient study. The tube voltage was kept constant at 120 kV and only the tube current time product was varied to achieve three different absorbed doses D_bl, CT_ = D1 (60 mAs), D_bl, CT_ = D2 (110 mAs) and D_bl, CT_ = D3 (180 mAs). For the determination of the number of individual baseline foci, one pair of samples was not irradiated (D0).

To mimic the patient study, the isolation of the leukocytes from the blood samples started 20 min after the irradiation in the CT.

#### 4.3.2. Absorbed Dose Measurements

A 3D-printed blood phantom was constructed to fit exactly in the round bottom tubes (Sarstedt) used for the irradiation of the blood samples. The density of the material (polyamide PA 12; 1.01 g cm^−3^) was chosen to be similar to the density of blood. In the middle of the phantom, a cavity was designed so that an ionization chamber (type 23323, PTW, Freiburg, Germany) could be positioned centrally in the tube. To ensure a precise measurement, the ionization chamber was first irradiated with a high tube current time product (240 mAs), the low-dose-setting (0.5 to 58 mGy) was chosen and a zero adjustment was performed before each experiment. For every irradiation process, the tube with the blood phantom and the ionization chamber was placed at a defined position on the patient bench next to the two blood samples. The dose value (D_bl, CT_) displayed on a calibrated dosimeter (UNIDOS, PTW) connected to the ionization chamber was noted for every irradiation process.

### 4.4. Evaluation of DNA Damage

The processing of the blood samples and the evaluation of co-localizing γ-H2AX+53BP1 DSB foci followed the protocol described by Eberlein et al. [9]. Briefly, leukocytes (more precisely: peripheral blood mononuclear cells) were separated by density centrifugation in BD Vacutainer CPT tubes (BD, Heidelberg, Germany), twice washed with PBS, fixed in 70% ethanol and stored at −20 °C. Immunofluorescence staining and DSB foci analysis [42] was performed at the Bundeswehr Institute of Radiobiology in Munich, Germany.

In each sample, co-localizing γ-H2AX+53BP1 foci were counted in 100 cells by an experienced investigator (HS). The evaluation of co-localizing foci only was chosen since this avoids counting of false positive γ-H2AX foci and increases the accuracy of the assay [43]. To calculate the average number of radiation-induced DSB foci (RIF) per cell, the average number of the individual baseline foci per cell was subtracted from the average number of the counted foci per cell in the irradiated samples.

### 4.5. Statistical Analysis

OriginPro 2017 (Origin Lab Corporation, Northampton, MA, USA) was used for plotting and analysis of data, and for statistical evaluation. The Shapiro-Wilk-test was used to test normality distribution. To test for differences between two dependent groups of normally distributed data, paired *t*-tests were performed. If normality was rejected, the Wilcoxon test was applied. To compare slopes of linear fits, Akaike information criteria (AIC) and an F-test were performed. Generally, results were considered significant for *p* < 0.05.

Assuming a Poisson-distribution, the standard deviation of the foci count was calculated. For the calculation of the standard deviation of the average number of RIF per cell, error propagation was performed, considering additionally the standard deviation of the baseline foci count. In general, standard deviations are given for all mean values. For fit parameters, standard errors are stated.

## 5. Conclusions

By correlating DNA damage in blood leukocytes quantified by using the γ-H2AX+53BP1 DSB focus assay with the absorbed doses to the blood after administration of [^68^Ga]Ga-PSMA I&T, we were able to show that even at very low absorbed doses to the blood of less than 3 mGy, the number of DSBs in the blood is significantly increased compared to baseline. The numbers were higher as expected from extrapolations of the results of previous studies that focused on a higher dose range. 

Whole-body CT that was performed in combination with the PET acquisition also induced a significant increase of RIF that was presumably related to a CT-induced absorbed dose of about 12 mGy. This indicates that, for this hybrid imaging scenario, the contribution of the CT to the absorbed dose to the blood is higher than that of the ^68^Ga, even though the DNA damage induction is comparable.

In conclusion, our results emphasize the need for more investigations on DSB induction and repair after internal irradiation in the very low dose range of diagnostic examinations. So far, there are only few studies with informative results in this area. This might be due to the fact that the very low dose range is difficult to assess and most biodosimetric assays are not sensitive enough to provide statistically significant results. The γ-H2AX+53BP1 focus assay, in contrast, proves as a useful tool for the quantification of radiation-induced DSBs even after internal exposures to absorbed doses of less than 10 mGy.

## Supplementary Materials

The following are available online at https://www.mdpi.com/2072-6694/12/2/388/s1: Table S1: Patient demographic, clinical and scan-related data.

## References

[1] Fendler W.P., Eiber M., Beheshti M., Bomanji J., Ceci F., Cho S., Giesel F., Haberkorn U., Hope T.A., Kopka K. (2017). ^68^Ga-PSMA PET/CT: Joint EANM and SNMMI procedure guideline for prostate cancer imaging: Version 1.0. Eur. J. Nucl. Med. Mol. Imaging.

[2] Herrmann K., Bluemel C., Weineisen M., Schottelius M., Wester H.J., Czernin J., Eberlein U., Beykan S., Lapa C., Riedmiller H. (2015). Biodistribution and radiation dosimetry for a probe targeting prostate-specific membrane antigen for imaging and therapy. J. Nucl. Med..

[3] Weineisen M., Schottelius M., Simecek J., Baum R.P., Yildiz A., Beykan S., Kulkarni H.R., Lassmann M., Klette I., Eiber M. (2015). ^68^Ga- and ^177^Lu-Labeled PSMA I&T: Optimization of a PSMA-Targeted Theranostic Concept and First Proof-of-Concept Human Studies. J. Nucl. Med..

[4] Rogakou E.P., Boon C., Redon C., Bonner W.M. (1999). Megabase chromatin domains involved in DNA double-strand breaks in vivo. J. Cell Biol..

[5] Rogakou E.P., Pilch D.R., Orr A.H., Ivanova V.S., Bonner W.M. (1998). DNA double-stranded breaks induce histone H2AX phosphorylation on serine 139. J. Biol. Chem..

[6] Schultz L.B., Chehab N.H., Malikzay A., Halazonetis T.D. (2000). p53 binding protein 1 (53BP1) is an early participant in the cellular response to DNA double-strand breaks. J. Cell Biol..

[7] Schumann S., Scherthan H., Lapa C., Serfling S., Muhtadi R., Lassmann M., Eberlein U. (2019). DNA damage in blood leucocytes of prostate cancer patients during therapy with ^177^Lu-PSMA. Eur. J. Nucl. Med. Mol. Imaging.

[8] Eberlein U., Scherthan H., Bluemel C., Peper M., Lapa C., Buck A.K., Port M., Lassmann M. (2016). DNA Damage in Peripheral Blood Lymphocytes of Thyroid Cancer Patients after Radioiodine Therapy. J. Nucl. Med..

[9] Eberlein U., Nowak C., Bluemel C., Buck A.K., Werner R.A., Scherthan H., Lassmann M. (2015). DNA damage in blood lymphocytes in patients after ^177^Lu peptide receptor radionuclide therapy. Eur. J. Nucl. Med. Mol. Imaging.

[10] Eberlein U., Peper M., Fernandez M., Lassmann M., Scherthan H. (2015). Calibration of the gamma-H2AX DNA double strand break focus assay for internal radiation exposure of blood lymphocytes. PLoS ONE.

[11] Schumann S., Eberlein U., Muller J., Scherthan H., Lassmann M. (2018). Correlation of the absorbed dose to the blood and DNA damage in leukocytes after internal ex-vivo irradiation of blood samples with Ra-224. EJNMMI Res..

[12] Schumann S., Eberlein U., Muhtadi R., Lassmann M., Scherthan H. (2018). DNA damage in leukocytes after internal ex-vivo irradiation of blood with the alpha-emitter Ra-223. Sci. Rep..

[13] Lassmann M., Hanscheid H., Gassen D., Biko J., Meineke V., Reiners C., Scherthan H. (2010). In vivo formation of gamma-H2AX and 53BP1 DNA repair foci in blood cells after radioiodine therapy of differentiated thyroid cancer. J. Nucl. Med..

[14] Rief M., Hartmann L., Geisel D., Richter F., Brenner W., Dewey M. (2018). DNA double-strand breaks in blood lymphocytes induced by two-day ^99m^Tc-MIBI myocardial perfusion scintigraphy. Eur. Radiol..

[15] Lee W.H., Nguyen P., Hu S., Liang G., Ong S.G., Han L., Sanchez-Freire V., Lee A.S., Vasanawala M., Segall G. (2015). Variable activation of the DNA damage response pathways in patients undergoing single-photon emission computed tomography myocardial perfusion imaging. Circ. Cardiovasc. Imaging.

[16] Okuda K., Watanabe N., Hashimoto M., Doai M., Kawai Y., Takahashi T., Arikawa T., Ooiso K., Sunatani Y., Iwabuchi K. (2019). Preliminary quantitative evaluation of radiation-induced DNA damage in peripheral blood lymphocytes after cardiac dual-isotope imaging. Appl. Radiat. Isot..

[17] May M.S., Brand M., Wuest W., Anders K., Kuwert T., Prante O., Schmidt D., Maschauer S., Semelka R.C., Uder M. (2012). Induction and repair of DNA double-strand breaks in blood lymphocytes of patients undergoing ^18^F-FDG PET/CT examinations. Eur. J. Nucl. Med. Mol. Imaging.

[18] Schnarr K., Carter T.F., Gillis D., Webber C., Lemon J.A., Dayes I., Dolling J.A., Gulenchyn K., Boreham D.R. (2015). Biological Response of Positron Emission Tomography Scan Exposure and Adaptive Response in Humans. Dose Response.

[19] Prasad A., Visweswaran S., Kanagaraj K., Raavi V., Arunan M., Venkatachalapathy E., Paneerselvam S., Jose M.T., Ozhimuthu A., Perumal V. (2019). ^18^F-FDG PET/CT scanning: Biological effects on patients: Entrance surface dose, DNA damage, and chromosome aberrations in lymphocytes. Mutat. Res..

[20] Nautiyal A., Mondal T., Mukherjee A., Mitra D., Kaushik A., Goel H.C., Goel A., Dey S.K. (2019). Quantification of DNA damage in patients undergoing non-contrast and contrast enhanced whole body PET/CT investigations using comet assay and micronucleus assay. Int. J. Radiat. Biol..

[21] Beels L., Werbrouck J., Thierens H. (2010). Dose response and repair kinetics of gamma-H2AX foci induced by in vitro irradiation of whole blood and T-lymphocytes with X- and gamma-radiation. Int. J. Radiat. Biol..

[22] Beels L., Bacher K., De Wolf D., Werbrouck J., Thierens H. (2009). gamma-H2AX foci as a biomarker for patient X-ray exposure in pediatric cardiac catheterization: Are we underestimating radiation risks?. Circulation.

[23] Beels L., Bacher K., Smeets P., Verstraete K., Vral A., Thierens H. (2012). Dose-length product of scanners correlates with DNA damage in patients undergoing contrast CT. Eur. J. Radiol..

[24] Vandevoorde C., Franck C., Bacher K., Breysem L., Smet M.H., Ernst C., De Backer A., Van De Moortele K., Smeets P., Thierens H. (2015). gamma-H2AX foci as in vivo effect biomarker in children emphasize the importance to minimize x-ray doses in paediatric CT imaging. Eur. Radiol..

[25] Waldren C.A. (2004). Classical radiation biology dogma, bystander effects and paradigm shifts. Hum. Exp. Toxicol..

[26] Rothkamm K., Löbrich M. (2003). Evidence for a lack of DNA double-strand break repair in human cells exposed to very low x-ray doses. Proc. Natl. Acad. Sci. USA.

[27] Grudzenski S., Raths A., Conrad S., Rübe C.E., Löbrich M. (2010). Inducible response required for repair of low-dose radiation damage in human fibroblasts. Proc. Natl. Acad. Sci. USA.

[28] Lengert N., Mirsch J., Weimer R.N., Schumann E., Haub P., Drossel B., Lobrich M. (2018). AutoFoci, an automated high-throughput foci detection approach for analyzing low-dose DNA double-strand break repair. Sci. Rep..

[29] Harbron R., Ainsbury E.A., Bouffler S.D., Tanner R.J., Eakins J.S., Pearce M.S. (2017). Enhanced radiation dose and DNA damage associated with iodinated contrast media in diagnostic X-ray imaging. Br. J. Radiol..

[30] Jost G., Golfier S., Pietsch H., Lengsfeld P., Voth M., Schmid T.E., Eckardt-Schupp F., Schmid E. (2009). The influence of X-ray contrast agents in computed tomography on the induction of dicentrics and gamma-H2AX foci in lymphocytes of human blood samples. Phys. Med. Biol..

[31] Grudzenski S., Kuefner M.A., Heckmann M.B., Uder M., Löbrich M. (2009). Contrast medium-enhanced radiation damage caused by CT examinations. Radiology.

[32] Pathe C., Eble K., Schmitz-Beuting D., Keil B., Kaestner B., Voelker M., Kleb B., Klose K.J., Heverhagen J.T. (2011). The presence of iodinated contrast agents amplifies DNA radiation damage in computed tomography. Contrast Media Mol. Imaging.

[33] Deinzer C.K., Danova D., Kleb B., Klose K.J., Heverhagen J.T. (2014). Influence of different iodinated contrast media on the induction of DNA double-strand breaks after in vitro X-ray irradiation. Contrast Media Mol. Imaging.

[34] Gould R., McFadden S.L., Horn S., Prise K.M., Doyle P., Hughes C.M. (2016). Assessment of DNA double-strand breaks induced by intravascular iodinated contrast media following in vitro irradiation and in vivo, during paediatric cardiac catheterization. Contrast Media Mol. Imaging.

[35] Piechowiak E.I., Peter J.F., Kleb B., Klose K.J., Heverhagen J.T. (2015). Intravenous Iodinated Contrast Agents Amplify DNA Radiation Damage at CT. Radiology.

[36] Wang L., Li Q., Wang X.M., Hao G.Y., Jie B., Hu S., Hu C.H. (2017). Enhanced radiation damage caused by iodinated contrast agents during CT examination. Eur. J. Radiol..

[37] Harbron R.W., Ainsbury E.A., Bouffler S.D., Tanner R.J., Pearce M.S., Eakins J.S. (2018). The impact of iodinated contrast media on intravascular and extravascular absorbed doses in X-ray imaging: A microdosimetric analysis. Phys. Med..

[38] Chronis F., Rogakou E.P., Gewirtz D.A., Holt S.E., Grant S. (2007). Interplay Between γH2AX and 53BP1 Pathways in DNA Double-Strand Break Repair Response. Apoptosis, Senescence, and Cancer. Cancer Drug Discovery and Development.

[39] Bolch W.E., Eckerman K.F., Sgouros G., Thomas S.R. (2009). MIRD pamphlet No. 21: A generalized schema for radiopharmaceutical dosimetry—Standardization of nomenclature. J. Nucl. Med..

[40] Eckerman K.F., Endo A. (2008). MIRD: Radionuclide Data and Decay Schemes.

[41] Stabin M.G., Sparks R.B., Crowe E. (2005). OLINDA/EXM: The second-generation personal computer software for internal dose assessment in nuclear medicine. J. Nucl. Med..

[42] Lamkowski A., Forcheron F., Agay D., Ahmed E.A., Drouet M., Meineke V., Scherthan H. (2014). DNA damage focus analysis in blood samples of minipigs reveals acute partial body irradiation. PLoS ONE.

[43] Rothkamm K., Barnard S., Moquet J., Ellender M., Rana Z., Burdak-Rothkamm S. (2015). DNA damage foci: Meaning and significance. Environ. Mol. Mutagen..

