# Supplementary material for: DNA Damage in Blood Leukocytes of Prostate Cancer Patients Undergoing PET/CT Examinations with [68Ga]Ga-PSMA I&T
Source: Cancers (Basel). 2020 Feb 7;12(2):388. doi: 10.3390/cancers12020388 (PMC7072291; doi:10.3390/cancers12020388)
Supplement: Supplementary file 1 [file cancers-12-00388-s001.pdf]

**Table 1.** Patient demographic, clinical and scan-related data.

| Patient ID | Age (years) | Weight (kg) | Adm. Activity (MBq) | Gleason Score | PSA level (ng $ml^{-1}$) | Indication | Pretreatments | $CTDI_{vol}$ (mGy)* |
|---|---|---|---|---|---|---|---|---|
| P1 | 70 | 79 | 158 | 4+4 | 10 | PSA-relapse | Local RTx (proton beam) | 13.45 |
| P2 | 73 | 80 | 147 | 4+5 | 0.3 | PSA-relapse | Surgery | 12.31 |
| P3 | 69 | 113 | 134 | 4+3 | 0.8 | PSA-relapse | Surgery + local RTx | 18.41 |
| P4 | 73 | 77 | 127 | 4+4 | 31 | re-staging | ADT, abiraterone, enzalutamide, docetaxel, cabazitaxel, RLT, PSMA-directed bispecific antibodies | 11.52 |
| P5 | 74 | 77 | 121 | 4+5 | 100 | progressive disease | RTx, docetaxel | 12.42 |
| P6 | 64 | 74 | 174 | 3+4 | 35 | primary staging | None | 13.10 |
| P7 | 73 | 90 | 110 | n/a | 0.2 | re-staging | Surgery, ADT | 14.49 |
| P8 | 72 | 80 | 121 | 3+3 | 36 | re-staging | None (watch & wait) | 11.95 |
| P9 | 67 | 118 | 121 | 4+5 | 33 | re-staging | ADT, RTx (lumbar spine), docetaxel, [$^{223}$Ra]$RaCl_2$, RLT | 20.15 |
| P10 | 56 | 100 | 163 | 4+4 | 12 | primary staging | None | 18.18 |
| P11 | 73 | 83 | 175 | 3+4 | 1.9 | PSA-relapse | Surgery + local RTx | 12.96 |
| P12 | 73 | 106 | 144 | 4+4 | 0.3 | PSA-relapse | Surgery | 17.48 |
| P13 | 69 | 87 | 143 | 4+4 | 1.0 | PSA-relapse | Surgery | 14.02 |
| P14 | 82 | 87 | 137 | n/a | 12 | PSA-relapse | High intensity focused ultrasound | 10.95 |
| P15 | 81 | 78 | 118 | 3+4 | n/a | re-staging | RTx | 12.66 |

* Here, only $CTDI_{vol}$ of the whole-body CT is listed. The $CTDI_{vol}$ related to the CT of the thorax was 6.1 mGy for all patients.

ADT = androgen deprivation therapy; PSMA = prostate specific membrane antigen; RLT = radio ligand therapy (with $^{177}$Lu-labelled therapeutic PSMA targeting compounds); RTx = radiation therapy.
